# Transplant Trial Watch

**DOI:** 10.3389/ti.2024.13111

**Published:** 2024-04-26

**Authors:** Simon R. Knight, John M. O’Callaghan

**Affiliations:** ^1^ Oxford Transplant Centre, Churchill Hospital, Oxford, United Kingdom; ^2^ Centre for Evidence in Transplantation, Nuffield Department of Surgical Sciences, University of Oxford, Oxford, United Kingdom; ^3^ University Hospitals Coventry and Warwickshire, Coventry, United Kingdom

**Keywords:** heart transplantation, randomised controlled trial, systematic review, living kidney donation, hemodynamic instability

To keep the transplantation community informed about recently published level 1 evidence in organ transplantation ESOT and the Centre for Evidence in Transplantation have developed the Transplant Trial Watch. The Transplant Trial Watch is a monthly overview of 10 new randomised controlled trials (RCTs) and systematic reviews. This page of Transplant International offers commentaries on methodological issues and clinical implications on two articles of particular interest from the CET Transplant Trial Watch monthly selection. For all high quality evidence in solid organ transplantation, visit the Transplant Library: www.transplantlibrary.com.

Randomised Controlled Trial 1 Use of Intraoperative Haemoadsorption in Patients Undergoing Heart Transplantation: A Proof-of-Concept Randomized Trial.

*by Nemeth, E., et al. ESC heart failure 2023 [record in progress]*.

## Aims

This study aimed to investigate the role of intraoperative haemoadsorption in orthotopic heart transplant patients.

## Interventions

Participants were randomised to receive either intraoperative haemoadsorption or standard care.

## Participants

60 patients undergoing orthotopic heart transplantation.

## Outcomes

The primary endpoint was early post-operative haemodynamic instability. Secondary endpoints were changes in procalcitonin (PCT) and C-reactive protein (CRP) levels post-operation, intraoperative change in mycophenolic acid (MPA) concentration, early allograft rejection, frequency of post-operative organ dysfunction, adverse immunological events, major complications, duration of ICU and in-hospital stay, and 1-year survival.

## Follow-Up

1 year.

## CET Conclusion

*by John O’Callaghan*

This is a very interesting, novel, RCT in heart transplantation. Heart recipients were randomised to standard care or to receive additional therapy with intra-operative hemoadsorption with the Cytosorb system from CytoSorbents, NJ, United States. The hemoadsorption cartridge was integrated into the cardiopulmonary bypass system and has been shown previously to remove cytokines, chemokines, bilirubin, myoglobin and plasma free haemoglobin. Patients were blinded to the treatment allocation, but clinical professionals were not. No sample size calculation could be done due to a lack of prior data on which to base it. The study found statistically significant differences across a range of outcomes, including the primary outcomes. Patients receiving hemoadsorption had a lower vasoactive-inotropic score, frequency of vasoplegic syndrome, risk of AKI, shorter median mechanical ventilation and median intensive care stay (by 3.5 days). The rates of cardiac allograft rejection, 30-day mortality and 1-year survival were similar between the groups, although it may have been too small to show differences in these outcomes. There were no device related complications.

## Jadad Score

3.

## Data Analysis

Modified intention-to-treat analysis.

## Allocation Concealment

Yes.

## Trial Registration

ClinicalTrials.gov—NCT03145441.

## Funding Source

No funding received.

**Systematic Review**

Psychological Impact of Living Kidney Donation: A Systematic Review by the EAU-YAU Kidney Transplant Working Group.

*by Cazauvieilh, V., et al. Transplant International 2023; 36: 11827.*

## Aims

This study aimed to examine the psychological effects of donating a kidney on living donors.

## Interventions

A literature search was performed using Pubmed and Medline. Study screening and data extraction were performed by two independent reviewers. The ROBINS-I tool was used to assess the risk of bias.

## Participants

23 studies were included in the review.

## Outcomes

The main outcomes of interest included assessment of quality of life, anxiety/depression, regret of donation, psychological impact over failure of transplant/death, and consequence of donation on donor/recipient relationship.

## Follow-Up

N/A.

## CET Conclusion

*by John O’Callaghan*

This is an interesting, well-conducted, and well-written, systematic review in living donation that gives a good description of the complexity in the donor-recipient relationship and the psychological outcome for the donor. Two independent reviewers screened references, extracted data and performed the risk-of bias assessment, which is clearly presented. A broad search was done, albeit only within pubmed/medline. 23 studies were included, comprised of a total 2,732 donors. The authors give a detailed description of the studies in narrative review. There is quantitative evidence from 3 studies that quality of life is the same pre and post-donation, whilst another 4 studies found quantitative evidence of improved quality of life at 1 year post-donation. These studies indicate risk factors that may be predictive of decreased donor quality of life such as donor fatigue, anxiety, depression, lack of social support, the donor-recipient relationship and any complications for the recipient. Three studies found no evidence of an impact of socio-economic status on quality-of-life post-donation. In general, studies found that the relationship between donors and recipients remained unchanged or improved/became closer. Some donors expected that their role as a carer for the recipient would decrease after donation. If this did not happen, donors felt disappointed or frustrated. In the majority of cases, donors were satisfied and did not regret donation. Importantly it was clearly demonstrated that it was possible to regret donation oneself, but to still recommend it for others. All studies showed a low rate of regret. There was some evidence of correlation between regret and the recipient’s outcome from the transplant, but evidence was conflicting. One interesting complexity highlighted by the study is that donors used conscious or unconscious strategies to influence the transplant team to select them as a donor. This may make it difficult to interpret the results of pre and post-donation comparisons. The authors also acknowledge the impact of social desirability bias, which may have affected donor responses to questionnaires.

## Trial Registration

N/A.

## Funding Source

Not reported.

## Clinical Impact Summary

*by Simon Knight*

Whilst the medical consequences of living kidney donation are largely understood through use of large-scale registry data, the psychosocial response to donor assessment and donation are less comprehensively documented. A wide variety of qualitative and quantitative approaches have been taken, often with conflicting findings. Previous systematic reviews have focussed mainly on qualitative studies using questionnaires to assess quality of life, anxiety and depression [1]. In an attempt to make more sense of the existing literature, working group of young academics from the European Association of Urology have undertaken a detailed systematic review of both qualitative and quantitative studies reporting the psychosocial impact of living kidney donation [2].

The group identified 8 qualitative and 15 quantitative studies, and due to heterogeneity in the instruments used undertook narrative analysis of the findings. Whilst quantitative studies demonstrated stable or improved quality of life with low levels of regret, the more detailed exploration afforded by qualitative approaches demonstrated a much more mixed, complex picture. Donation can often impact quality of life, particularly in donors that experience post-operative fatigue, and many donors experience post-operative anxiety and depression with some expressing a sense of abandonment following donation. These aspects seem particularly important in the presence of donor or recipient medical complications, highlighting the importance of regular follow-up in donors. Despite this, very few donors express regret and most would recommend the process.

An interesting aspect that comes out of the qualitative studies is the impact of the pre-donation phase, with some donors describing anxiety induced by the investigations and work-up process, in particular relating to the fear of being found unsuitable, and the length of the process. Some donors reported employing strategies to influence decisions, such as downplaying existing psychological illnesses and withholding medical information to improve their chances of being found suitable to donate. Again, this highlights this importance of a detailed workup for all donors, including psychological assessment where indicated by history or clinical concerns.

One limitation of the existing literature is that it is difficult to identify those subgroups most at risk of psychological complications from the donation process. A few studies report the impact of recipient complications, donor-recipient relationship or social support on outcomes, but data on other aspects such as donor age (particular younger donors) and donor complications are lacking.

Overall, this review is a well-conducted study that provides a very comprehensive summary of what we currently know about the psychosocial impact of living donation. It also helps to highlight areas for future research.

## Clinical Impact

3/5.
